# Diagnostic models for sepsis-associated encephalopathy: a comprehensive systematic review and meta-analysis

**DOI:** 10.3389/fneur.2025.1645397

**Published:** 2025-07-31

**Authors:** Tengfei Zhou, Xinming Tian, Wei Wang, Zhe Chu

**Affiliations:** ^1^Department of Emergency, The First Hospital of Jilin University, Changchun, Jilin, China; ^2^School of Nursing, Jilin University, Changchun, Jilin, China

**Keywords:** sepsis-associated encephalopathy, prediction, model, systematic review, meta-analysis

## Abstract

**Objective:**

To systematically evaluate the performance and methodological rigor of published prediction models for sepsis-associated encephalopathy (SAE), identify their limitations, and provide guidance for the future development of robust and clinically applicable models.

**Methods:**

We conducted a systematic search across nine English and Chinese databases (from inception to May 2025) for studies developing or validating SAE prediction models in adult sepsis patients. Two researchers independently gathered data, using PROBAST to assess methodological quality, and conducted a meta-analysis of the AUC of logistic regression models.

**Results:**

Ten studies were included, encompassing 55,244 patients with sepsis, revealing an incidence of SAE ranging from 15.0 to 62.4%. A total of 29 predictive models were developed, comprising 10 optimal models, primarily utilizing logistic regression or machine learning algorithms. The combined AUC of the five logistic regression models was 0.85 (95% CI 0.77–0.93), exhibiting substantial heterogeneity (I^2^ = 91.8%). All models showed a high risk of bias according to the PROBAST evaluation, mainly due to the lack of external validation and methodological shortcomings.

**Conclusion:**

Current SAE prediction models demonstrate moderate discriminatory ability, but their methodological quality remains poor, and they are not yet suitable for routine clinical application. In the future, standardized SAE definitions and prospective data collection should be strengthened, models should be developed and validated strictly following the TRIPOD guidelines, and model interpretability should be improved to promote clinical application.

**Systematic review registration:**

https://www.crd.york.ac.uk/PROSPERO/view/CRD420251062747.

## 1 Introduction

Sepsis-associated encephalopathy (SAE) refers to widespread brain dysfunction caused by sepsis without central nervous system infection, which requires the identification and exclusion of other encephalopathies as a prerequisite (1). Research indicates that SAE is the predominant form of critical encephalopathy in intensive care units (ICUs) (2, 3). The pathophysiology of SAE is highly complex, clinical diagnosis remains challenging, and the absence of standardized diagnostic criteria leads to significant variability in its occurrence among research (4, 5). A retrospective cohort study from Chinese ICUs indicated an SAE incidence of 48.1% (6), whereas French ICUs reported an incidence of 53.0% (7), and the United States Critical Care Database recorded approximately 70.0% (8). SAE may clinically present as agitation, diminished attention, altered consciousness, delirium, and coma, all of which are closely associated with poor patient outcomes (9, 10). Previous studies have indicated that SAE significantly increases the mortality rate of patients within 30 days or even 1 year (11, 12). Furthermore, SAE may result in extended ICU admissions and enduring cognitive deficits in patients (13). Current studies have shown that the destruction of the blood–brain barrier (BBB) is one of the core factors in the pathogenesis of SAE (14, 15). Individuals with sepsis exhibit a systemic inflammatory response that results in the release of endotoxins and proinflammatory cytokines. These substances stimulate brain endothelial cells and microglia, resulting in the downregulation of tight junction proteins and heightened blood–brain barrier permeability, permitting the ingress of inflammatory agents and toxins into the central nervous system, and inciting neuroinflammation and cerebral dysfunction (16, 17). The diagnosis of SAE remains primarily clinical, often relying on delirium screening tools such as the Confusion Assessment Method for the Intensive Care Unit (CAM-ICU) or neurological assessment scales such as the Glasgow Coma Scale (GCS) (18, 19). However, these methods are highly subjective and lack sensitivity, and early identification remains challenging. Timely diagnosis of SAE can help patients develop personalized treatment plans, avoid risk factors that aggravate SAE as much as possible, and implement neuroprotective measures.

With the development of precision medicine, researchers have paid more attention to the early identification and risk stratification of SAEs. Many studies have developed predictive models to support clinical decision-making (20). These diagnostic models typically combine clinical characteristics, such as vital signs, organ dysfunction scores, and laboratory indicators, and some studies have incorporated specific biomarkers to assess the risk of SAE in patients with sepsis (21, 22). For example, Zhang et al. (23) explored the application of neuron-specific enolase (NSE) and regional cerebral oxygen saturation (rSO₂%) in SAE patients and found that elevated levels of both indicators were significantly associated with decreased 28-day survival rate (*p* < 0.001), suggesting that they have certain value in SAE risk stratification. Mowafy et al. (24) confirmed in a prospective study that the optic nerve sheath diameter (ONSD) of SAE patients was significantly widened (the critical value was 5.2 mm), supporting ONSD as a non-invasive, bedside early screening method, and proposed a multimodal evaluation strategy integrating clinical examination, imaging and ultrasound, which is expected to optimize the SAE management process. In addition, several studies have employed either traditional logistic regression or advanced machine learning algorithms to develop visualized prediction models for the diagnosis of SAE (25, 26). However, none of these models or predictors have been widely implemented in clinical settings, and considerable variation exists in terms of model performance, predictor selection, and validation approaches. Furthermore, concerns regarding methodological rigor and risk of bias have limited confidence in the clinical utility of these models.

To date, there is still a lack of a comprehensive systematic review of the integration and quality assessment of existing SAE prediction models. This study aims to systematically review the current status of SAE prediction models, summarize their construction process, core variables and performance, use the internationally recognized PROBAST tool (27) to evaluate their methodological quality, and point out some common problems in current research and areas that may need to be optimized in the future. The innovation of this study is that it is the first time to comprehensively evaluate the modeling methods, performance differences and bias risks of SAE prediction models based on systematic review and meta-analysis methods, including the comparison of the interpretability of common algorithms such as logistic regression, random forest, and XGBoost, and explore issues such as model external validation, variable screening, model visualization and clinical transformation. We hope that this study will provide clinical medical staff with a systematic understanding of SAE prediction tools, and also provide certain theoretical support and methodological references for the subsequent development of higher-quality and popularizable SAE early identification models.

## 2 Method

This study was conducted under the Preferred Reporting Items for Systematic Reviews and Meta-Analyses (PRISMA 2020) guidelines (28). The study protocol was registered in PROSPERO (CRD420251062747).

### 2.1 Search strategy

A thorough literature review was conducted to find research that developed or validated a predictive model for diagnosing SAE. The search strategy included Chinese (CNKI, VIP, Wanfang, CBM) and English (PubMed, Embase, Web of Science, Cochrane Library, CINAHL) electronic databases (from inception to May 1, 2025), using keywords such as “sepsis-associated encephalopathy,” “sepsis-associated delirium,” “nomogram,” “predict,” “prediction models,” “risk score,” and “predictors.” In the process of searching Chinese databases, we used Chinese keywords corresponding to the meanings of the English search terms and adjusted the search strategy appropriately according to the characteristics of each Chinese database. At the same time, we also consulted two experts in the field of critical care medicine to ensure the professionalism and coverage of the search expressions. In addition, we also conducted supplementary searches by reviewing the reference lists of the included articles to ensure that relevant articles were not missed as much as possible. The detailed search strategy can be found in the Supplementary material. We used PICOTS terminology to help construct the purpose of this study and the inclusion and exclusion criteria of the studies (29). The details are as follows:

- P (Population): Adult patients with sepsis-associated encephalopathy (SAE).
- I (Intervention): Developed or validated sepsis-associated encephalopathy (SAE) risk prediction models (including ≥ 2 predictors).
- C (Comparator): Not applicable.
- O (Outcome): Prediction of the occurrence of sepsis-associated encephalopathy (SAE) (including its diagnostic accuracy, such as AUC, sensitivity, and specificity), as well as model calibration and clinical utility.
- T (Timing): Basic information, clinical rating scale results, and laboratory indicators are collected after hospital admission to evaluate and predict the occurrence of SAE.
- S (Setting): The model aims to forecast the probability of SAE in patients with sepsis and help clinical medical staff take intervention measures before SAE occurs to improve patient prognosis.

### 2.2 Eligibility criteria

#### 2.2.1 Inclusion criteria

(1) Adult patients (>18 years) with sepsis; (2) Observational study design (cohort study, case–control study, cross-sectional study); (3) The study was to develop an SAE diagnostic model that contained two or more predictors; and (4) The primary outcome of the study is the occurrence of SAE, defined by clear diagnostic criteria (CAM-ICU, GCS, medical staff’s diagnosis).

#### 2.2.2 Exclusion criteria

(1) Only risk factors were analyzed, but no model was built, or model evaluation indicators were missing; (2) literature not published in English or Chinese; and (3) studies for which the full text was not available.

### 2.3 Study screening and data extraction

The search records were imported into NoteExpress software to remove duplicate records. Two researchers meticulously evaluated the literature according to the inclusion and exclusion criteria. When it was difficult to make a decision, the opinions of the third researcher were included until a consensus was reached.

Subsequently, an Excel data extraction table was constructed following the CHARMS checklist from the systematic review of predictive model research (29). We divided the extracted information into two categories: (1) Essential information: author, year of publication, study design, total sample size, SAE incidence, data source, SAE diagnostic criteria, candidate predictors, and final predictors included. (2) Model parameters: treatment methods for missing values and continuous variables, variable screening methods, model building methods, model performance, model verification, model clinical benefits, and final model presentation. Data extraction was completed independently by two researchers. When there was a disagreement, the opinions of the third researcher were included until a consensus was reached.

### 2.4 Quality assessment

Two authors independently assessed the methodological quality of each study using the PROBAST scale, and any disagreements were resolved through consultation with a third author. PROBAST examined four domains: “participants” (2 signal questions), “predictors” (3 signal questions), “predicted outcomes” (6 signal questions), and “analysis” (9 signal questions). Structured risk of bias (ROB) judgments were made across four domains. The answers to each signal question included “yes,” “probably yes,” “no,” “probably no,” or “unclear,” and the answers were used to determine the ROB of each domain and the overall ROB of the target prediction model (low, high, or unclear). Each study was assessed for overall risk of bias (if any domain was rated as high risk, the overall ROB was “high”).

### 2.5 Statistical analysis

Given the large differences in modeling methods used by different studies, especially in algorithm type, feature selection process, and verification method, this study mainly used descriptive analysis methods to summarize the basic characteristics, model parameters, bias risks, and applicability evaluation of the included studies (assessed using the PROBAST tool). To ensure the comparability and robustness of the meta-analysis, we only included models that used logistic regression algorithms and reported the area under the receiver operating characteristic curve (AUC) for meta-analysis. In contrast, the data of the models constructed by machine learning all came from the MIMIC database, and there was a problem of sample overlap between studies. If included in the meta-analysis, it may lead to repeated statistics and increased bias, affecting the effectiveness of the analysis. In addition, we gave priority to models with better parameter performance. Stata software (version 17.0) was used to combine the AUC values of the models that met the criteria and draw a forest plot. The heterogeneity of the studies was assessed using the I^2^ index and the Cochrane Q test. When the I^2^ value exceeded 50%, significant heterogeneity was considered. We selected the random effects model for data pooling; otherwise, the fixed effects model was used. Sensitivity analysis was performed using the one-by-one elimination method to explore the sources of heterogeneity. We presented the predictors with higher frequencies in the model in the form of a bar graph. In addition, the possibility of publication bias was assessed using the Egger test, and a *p*-value > 0.05 indicated that the model was low in publication bias.

## 3 Results

### 3.1 Study selection and characteristics

**Figure 1:**
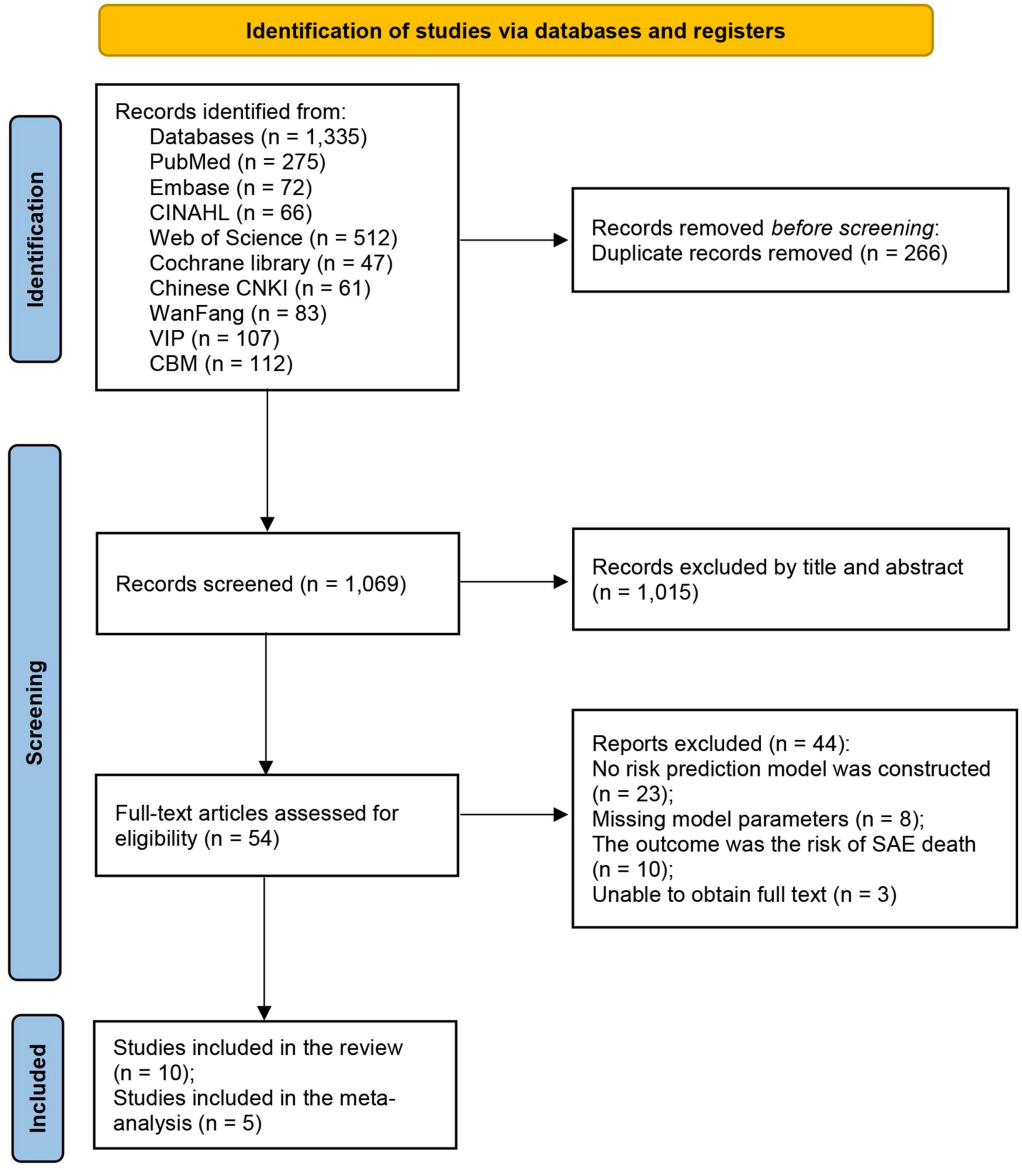
Detailed screening process for the study.

The detailed literature selection process of this study can be seen in Figure 1. A total of 1,335 articles were obtained in the initial search. After independent screening by two reviewers, the remaining 54 studies needed to be read in full text. Finally, 10 articles were included in this review in strict accordance with the inclusion and exclusion criteria (25, 26, 30–37).

Table 1 shows the essential information for the included studies. The publication years were from 2021 to 2025, including 8 English articles and 2 Chinese articles, but the authors of the English literature were all from China. All included studies were cohort studies: 8 of them were retrospective analyses, and 2 were prospective observational studies. The total sample size of the 10 studies was 55,244 patients with sepsis, of whom approximately 24,951 (approximately 45%) developed SAE. The sample sizes varied widely among the studies, ranging from 67 to 22,361 patients. Data sources included large critical care databases (six of the studies used the MIMIC-III or MIMIC-IV ICU database) and single-center hospital cohort studies (four of the studies). In the model constructed based on the MIMIC database, the average incidence of SAE was 48.8%, while in the Chinese single-center data model, the average incidence of SAE was 41.2%.

**Table 1:** Summary of included studies on prediction models for SAE.

Author (year)	Study design	SAE cases/total sample size (%)	Data source	SAE diagnostic criteria	Candidate predictors (*n*)	Final predictors included (*n*)
Zhao et al. (2021) (24)	Retrospective cohort	841/2,028 (41.5%) 214/507 (42.2%)	MIMIC III	GCS < 15, delirium, cognitive impairment, and medicating with haloperidol (ICD-9)	89	9 (age, qSOFA, quinolone antibiotics, carbapenem antibiotics, midazolam, diphenhydramine hydrochloride, heparin sodium injection, steroids, and H_2_-antagonist)
Ge et al. (2022) (25)	Retrospective cohort	6,284/12,460 (50.4%)	MIMIC III	GCS ≤ 14, subacute delirium, and delirium due to conditions classified elsewhere (ICD-9)	52	7 (age, GCS score, glucose, MAP, heart rate, hemoglobin, and ICU LOS)
Lu et al. (2022) (26)	Retrospective cohort	4,684/8,935 (52.4%)	MIMIC IV	GCS ≤ 14, delirium (ICD-9)	56	9 (age, SAPSII score, creatinine, resp. rate mean, PH, chlorine, platelet, phosphorus, and sodium)
Wang et al. (2023) (28)	Retrospective cohort	97/640 (15.2%) 45/300 (15.0%)	One tertiary hospital (January 2017–December 2021)	GCS < 15, delirium	39	5 (age, SpO2, S100β, albumin, use of vasopressors)
Zhou et al. (2023) (29)	Retrospective cohort	84/213 (39.4%)	One tertiary hospital (March 2021–February 2023)	CAM-ICU positive	27	7 (SOFA score, APACHE II score, ALT, albumin, rScO_2_, lactate level, PI)
Zhao et al. (2023) (27)	Retrospective cohort	8,290/22,361 (37.1%)	MIMIC IV	GCS < 15, delirium	20	5 (age, SOFA score, heart rate, sodium, temperature)
Mei et al. (2024) (19)	Prospective observational	32/67 (47.8%)	One hospital (January 2019–August 2023)	GCS < 15, CAM-ICU positive, medical staff diagnosis	19	3 (CCT, PI, S100β)
Jin et al. (2024) (30)	Retrospective cohort	2,781/4,476 (62.1%)	MIMIC IV	GCS < 15, delirium	43	9 (age, BMI, gender, SOFA score, MAP, sodium, platelet, temperature, midazolam)
Zhang et al. (2024) (31)	Prospective observational	63/101 (62.4%)	One hospital (January 2020–January 2022)	CAM-ICU positive	42	5 (age, neutrophils, CSF BNIP3 L, S100β, and ONSD)
Han et al. (2025) (20)	Retrospective cohort	1,536/3,156 (48.7%)	MIMIC IV	GCS < 15, delirium	45	18 (MAP, RR, HTN, COPD, CKD, SOFA score, OASIS, SAPS II, Charlson, WBC, sodium, platelet, hematocrit, glucose, anion gap, PCO_2_, PTT, and BUN)

MIMIC-III/IV, Medical Information Mart for Intensive Care III/IV; GCS, Glasgow Coma Scale; qSOFA, quick Sequential Organ Failure Assessment; MAP, Mean Arterial Pressure; ICU LOS, ICU length of stay; SAPS II, Simplified Acute Physiology Score II; SpO₂, peripheral capillary oxygen saturation; S100β, S100 calcium-binding protein beta; CAM-ICU, Confusion Assessment Method for the ICU; APACHE II, Acute Physiology and Chronic Health Evaluation II; ALT, alanine aminotransferase; rScO₂, regional cerebral oxygen saturation; PI, pulsatility index of the middle cerebral artery; CCT, cerebral circulation time; BMI, body mass index; ONSD, optic nerve sheath diameter; CSF, cerebrospinal fluid; BNIP3L, Bcl-2/adenovirus E1B 19 kDa-interacting protein 3-like; RR, respiratory rate; HTN, hypertension; COPD, chronic obstructive pulmonary disease; CKD, chronic kidney disease; OASIS, Oxford Acute Severity of Illness Score; Charlson, Charlson Comorbidity Index; WBC, White blood cell count; PCO₂, partial pressure of carbon dioxide; PTT, partial thromboplastin time; BUN, blood urea nitrogen.

**Figure 2:**
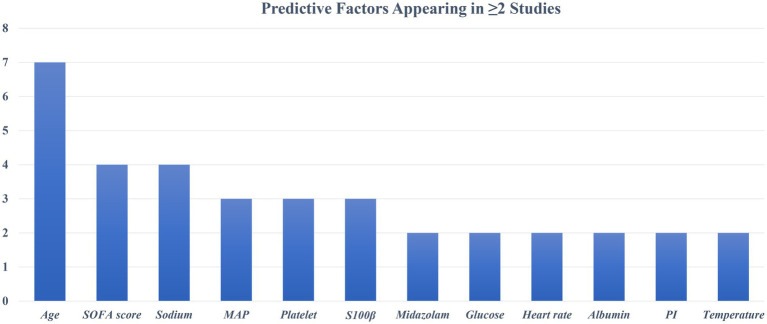
Frequency statistics of predictors in the model.

The diagnostic criteria for SAE are not completely uniform. Most studies define SAE as GCS values below a specific threshold (≤14 or <15) with evidence of delirium or acute altered mental status due to sepsis. Some studies have used different assessment methods. For example, Zhou et al. (35) and Zhang et al. (37) required a positive CAM-ICU test in patients with sepsis as an indicator of SAE. Mei et al. (25) defined SAE as a combination of GCS < 15, positive CAM-ICU, and clinical judgment of medical staff. Despite differences in assessments across studies, all aimed to identify acute encephalopathy changes associated with sepsis. The incidence of SAE in each study cohort ranged from as low as 15% (34) to more than 60% (36, 37). The maximum number of candidate predictors in the model is 89 (30), and the minimum is 19 (25) (Table 1). These included demographic characteristics, clinical severity scores, vital signs, laboratory parameters, and comorbidities. The most common predictor was age (*n* = 7, 70.0%), followed by serum sodium level (*n* = 4, 40.0%) and SOFA score (*n* = 4, 40.0%). Two prospective studies also evaluated neurological or biomarker predictors. Mei et al. (25) used brain injury biomarkers S100β and cerebral circulation time (CCT) for prediction; Zhang et al. (37) used CSF BNIP3 L as a predictor. Retrospective database studies mainly relied on routinely collected variables. Figure 2 summarizes the predictors of the final model (≥2 occurrences).

### 3.2 Model development method and performance

Details of model development, validation, and performance of the included studies are shown in Table 2. A total of 29 prediction models were constructed in the 10 included studies. Most retrospective studies applied missing value imputation, whereas 4 studies did not report the methods used for handling missing data. Continuous variables were not transformed or categorized in any of the included models.

**Table 2:** Summary of model development and performance metrics for each study.

Author (year)	Missing data handling	Continuous variable processing	Feature selection method	Model development method	AUC (Best performing model)	Calibration assessment	Internal validation	External validation	Clinical utility	Model presentation format
Zhao et al. (2021) (24)	Mean imputation	Unprocessed	LASSO regression	Multivariable logistic regression	AUC (TC) = 0.743; AUC (VC) = 0.762	Calibration curve: good agreement	1,000 bootstrap (8:2 random split)	Not reported	DCA (TC): 10%-90; DCA (VC): 10–89%	Nomogram
Ge et al. (2022) (25)	Single imputation	Unprocessed	Not reported	Logistic regression, SVM, DTC, RF, GBM, MLP, XGBoost, LGBM	AUC (LGBM-TC) = 0.91; AUC (LGBM-VC) = 0.87	Calibration curve: good agreement	7:3 random split, 5-fold cross-validation	Not reported	Not reported	Not reported
Lu et al. (2022) (26)	Multiple imputation	Unprocessed	Not reported	GBDT, RF, LGBM, DT, SVM, XGBoost	AUC (XGBoost-TC) = 0.902; AUC (XGBoost-VC) = 0.884	Expert-based calibration assessment	7:2:1 random split, 10-fold cross-validation	Not reported	Not reported	SHAP plot
Wang et al. (2023) (28)	Not reported	Unprocessed	Univariable logistic regression	Multivariable logistic regression (forward)	AUC (TC) = 0.810; AUC (VC) = 0.813	Calibration curve: good fit; H-L test (*p* = 0.304, 0.172)	Temporal validation	Not reported	Not reported	Nomogram
Zhou et al. (2023) (29)	Not reported	Unprocessed	Univariable logistic regression	Multivariable logistic regression (stepwise)	AUC = 0.831	Calibration curve: good fit; H-L test (*p* = 0.616)	Bootstrap	Not reported	Not reported	Nomogram
Zhao et al. (2023) (27)	Not reported	Unprocessed	Univariable logistic regression; clinical judgment (expert-based)	Multivariable logistic regression	AUC (TC) = 0.802; AUC (VC) = 0.809	Calibration curve: good consistency	Bootstrap	Not reported	DCA: demonstrated clinical benefit	Nomogram
Mei et al. (2024) (19)	Not reported	Unprocessed	Univariable analysis	Multivariable logistic regression	AUC = 0.924	Calibration curve: high concordance; H-L test (*p* = 0.944)	1,000 bootstrap	Not reported	DCA range: 0–100%	Nomogram
Jin et al. (2024) (30)	Multiple imputation	Unprocessed	LASSO regression	Multivariable logistic regression	AUC (TC) = 0.751; AUC (VC) = 0.766	Calibration curve: good fit; H-L test (*p* = 0.1264, 0.3734)	7:3 random split	Not reported	DCA (TC): 13%-92; DCA (VC): 19–90%	Nomogram
Zhang et al. (2024) (31)	Not reported	Unprocessed	Univariable analysis	Logistic regression, Neural networks, DT, Naïve Bayes	AUC (logistic regression) = 0.928	Calibration curve: better accuracy	7:3 random split	Not reported	DCA: showed improved net benefit	Not reported
Han et al. (2025) (20)	KNN imputer	Unprocessed	LASSO regression; Boruta methods	XGBoost, CatBoost, LGBM, MLP, SVM	AUC (XGBoost) = 0.898; AUC (simplified XGBoost) = 0.858	Calibration curve: stable performance	Bootstrap (7:3 random split), 10-fold cross-validation	Not reported	DCA: significant clinical benefit	SHAP plot

TC, training cohort; VC, validation cohort; AUC, area under the receiver operating characteristic curve; DCA, decision curve analysis; H-L test, Hosmer–Lemeshow test; SVM, support vector machine; DTC, decision tree classifier; RF, random forest; GBM, gradient boosting machine; MLP, multi-layer perceptron; XGBoost, extreme gradient boosting; LGBM, light gradient boosting machine; GBDT, gradient boosting decision tree; DT, decision tree; SHAP, SHapley additive exPlanations; CatBoost, categorical boosting; KNN imputer, K-Nearest Neighbors Imputer.

#### 3.2.1 Feature selection and model building

Three studies used LASSO (least absolute shrinkage and selection operator) regression to screen out important predictors. Five studies used univariate analysis to screen predictors, and two studies did not report the predictor screening method. Logistic regression was the most commonly used modeling method, with seven studies using it as the final model. Four studies conducted extensive comparisons of machine learning algorithms. The models with the best final SAE prediction effects were XGBoost (*n* = 2), LightGBM (*n* = 1), and logistic regression (*n* = 1). All studies measured the discrimination of the models by AUC. The AUC of the best-performing models ranged from 0.743 to 0.928 (Table 2). Among the logistic regression models, Zhou et al. (35) and Zhang et al. (37) reported an AUC of 0.743 (training cohort) and 0.762 (validation cohort); Jin et al. (36) achieved an AUC of 0.751 (training set) and 0.766 (test set). Compared to other models, these exhibited slightly lower discrimination and overall performance. Among machine learning models, Ge et al. (31) and Lu et al. (32) reported AUCs on the validation set greater than 0.85, and Han et al. (26) achieved an AUC of 0.898 for the unsimplified model.

#### 3.2.2 Model validation

None of the included studies reported external validation and only used different forms of internal validation. Wang et al. (34) used time-stratified validation to build a model with patient data from 2017 to 2020 and validated it in a 2021 cohort. One study used a combination of random split, bootstrap resampling, and cross-validation for model validation; another study used random split and bootstrap resampling for validation. Two other studies used random split combined with cross-validation, and two studies used only random split. In addition, three studies used only bootstrap resampling for internal validation. Overall, most of the existing models lack true external validation.

#### 3.2.3 Model performance and final presentation

Calibration performance was evaluated in all included studies, and the final models generally demonstrated good calibration. Most studies presented calibration results using calibration curves or the Hosmer-Lemeshow (H-L) goodness-of-fit test. Five studies only reported good calibration curves; four studies reported a combination of calibration curves and H-L test results (*p* > 0.05); and one study verified the model calibration performance through clinical expert evaluation. In addition, six studies employed decision curve analysis (DCA) to evaluate the clinical utility of the models. The results consistently showed favorable net benefits. For example, Mei et al. (25) presented a DCA curve covering the 0–100% threshold interval, indicating that the model has good clinical application value at almost all reasonable risk thresholds. Finally, most studies (*n* = 8) presented their final models in a visualized form. Five studies developed nomograms; two machine learning models provided feature importance plots (SHAP diagrams). Two studies constructed models but did not present visualizations.

### 3.3 Risk of bias and quality assessment

Table 3 shows the results of our systematic evaluation of the included studies in the process of predictive modeling based on the PROBAST tool. Overall, all ten included studies had a high risk of bias. In the field of research subjects, two studies (35, 37) were rated as high risk of bias due to problems with the inclusion and exclusion criteria of the research subjects and the opaque enrollment process. In the field of predictors, two studies (25, 37) were rated as high risk because they used special variables that are difficult to obtain in routine clinical practice, making the actual application of the model difficult. In the field of outcomes, three studies (25, 34, 35) were rated as high risk because there were differences in the definition of outcomes and the subjective judgment of clinical medical staff was included in the outcome assessment process, which may introduce bias. Finally, in the field of analysis, all studies were rated as high risk of bias. The primary reasons included the absence of external validation, inadequate assessment of model calibration, improper handling of missing data, reliance on univariate analysis for variable selection in some studies, and incomplete reporting of model performance metrics.

**Table 3:** PROBAST results of the included studies.

Included studies	ROB				Applicability			Overall	
	Participants	Predictors	Outcome	Analysis	Participants	Predictors	Outcome	Rob	Applicability
Zhao et al. (2021) (24)	Low	Low	Low	High	Low	Low	Low	High	Low
Ge et al. (2022) (25)	Low	Low	Low	High	Low	Low	Low	High	Low
Lu et al. (2022) (26)	Low	Low	Low	High	Low	Low	Low	High	Low
Wang et al. (2023) (28)	Low	Low	High	High	Low	Low	Low	High	Low
Zhou et al. (2023) (29)	High	Low	High	High	High	Low	Low	High	High
Zhao et al. (2023) (27)	Low	Low	Low	High	Low	Low	Low	High	Low
Mei et al. (2024) (19)	Low	High	High	High	Low	High	Low	High	High
Jin et al. (2024) (30)	Low	Low	Low	High	Low	Low	Low	High	Low
Zhang et al. (2024) (31)	High	High	Low	High	High	High	Low	High	High
Han et al. (2025) (20)	Low	Low	Low	High	Low	Low	Low	High	Low

ROB, risk of bias.

**Figure 3:**
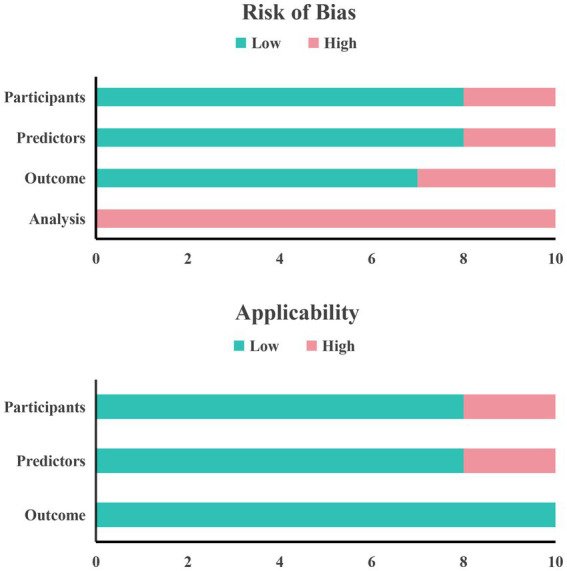
PROBAST tool model evaluation results for included studies.

In terms of applicability, three studies were rated as high risk. Among them, there are two studies in the field of research subjects that have certain limitations because the included research subjects do not fully match the definition of the problem of this study (35, 37). There are also two studies in the field of predictors whose definition and evaluation of predictor variables do not match the problem of this study (25, 37). In the field of outcomes, the definition, assessment and measurement of outcome indicators in the included studies met the requirements of this systematic review and the risk of bias in this area was assessed to be low. Finally, a summary bar graph was constructed to illustrate the risk of bias and applicability (Figure 3).

### 3.4 Meta-analysis of AUC

**Figure 4:**
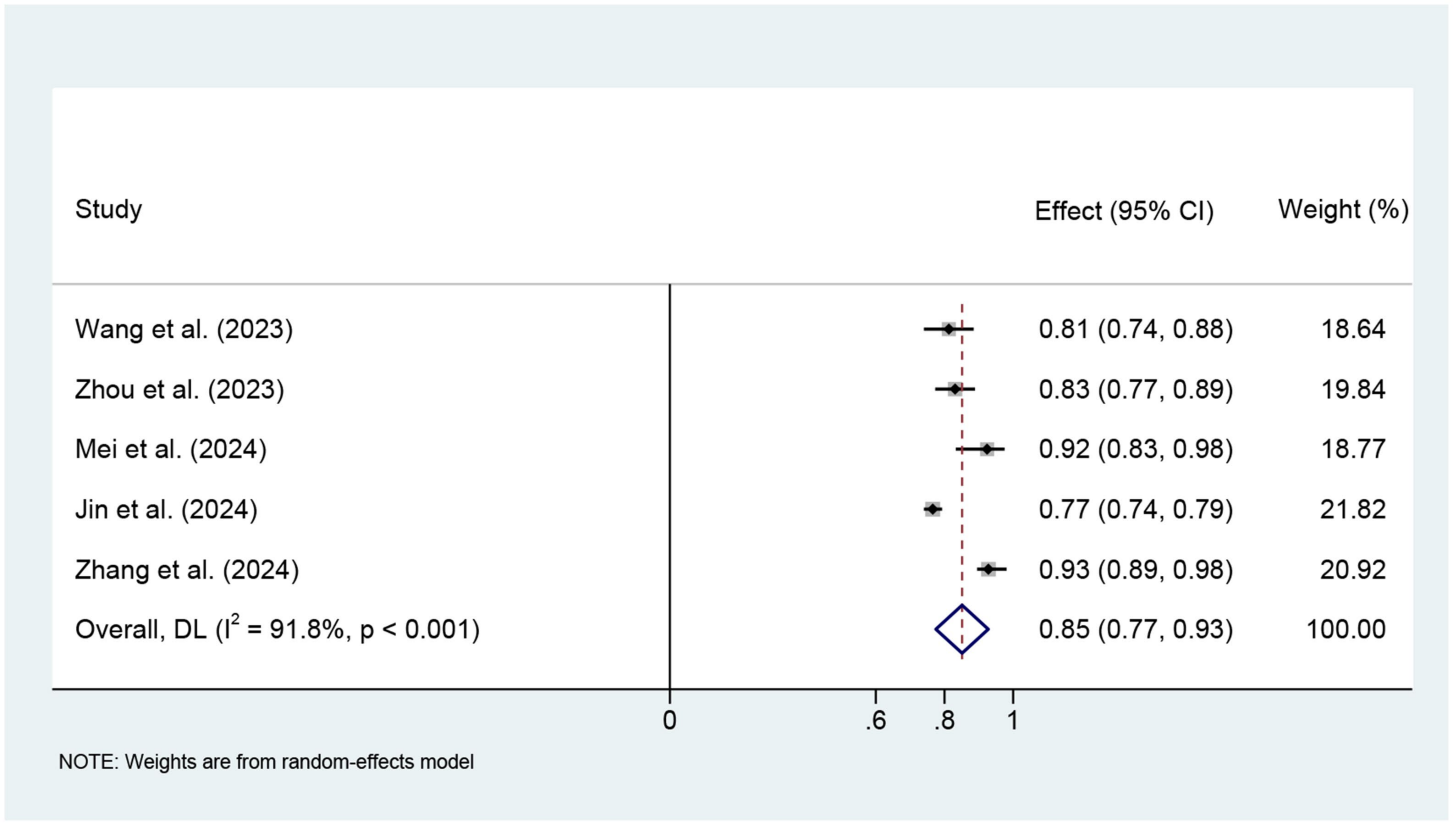
Meta-analysis forest plot of the combined AUC values of the 5 models.

We finally included five studies for meta-analysis using logistic regression models. The meta-analysis used a random effects model, and the combined AUC was 0.85 (95% CI: 0.77–0.93), with large heterogeneity (I^2^ = 91.8%, *p* < 0.001) (Figure 4). Sensitivity analysis showed that the study by Jin et al. (36) had the greatest impact on the combined AUC value (see Supplementary Figure 1 for details). After excluding this study, the combined AUC showed a significant decrease in I^2^ and an increase in AUC. The Egger test was used to assess publication bias, and the results showed no significant publication bias (t = 1.32, *p* = 0.279) (Supplementary Figure 2).

## 4 Discussion

SAE is one of the most common neurological complications of sepsis patients in the ICU and is closely related to the poor short-term and long-term prognosis of patients (38, 39). In this study, the incidence of SAE ranged from 15.0 to 62.4%, with a high overall level and significant differences. This difference may be related to the differences in data sources (US Critical Care Database and Chinese Single Center Hospital Data), patient population characteristics, and SAE assessment tools and diagnostic criteria. For example, the average age of the patients in the study by Wang et al. (34) was relatively low (57 years old), the prevalence of hypertension was 43.6%, the proportion of patients using vasopressors was 18.8%, and the incidence of SAEs was only 15.0%. In contrast, in the study by Jin et al. (36) based on the MIMIC database, the average age of the patients was 68 years old, the prevalence of hypertension was 63.6%, the proportion of patients using vasopressors was 64.2%, and the incidence of SAEs was as high as 62.1%. Patients of advanced age, those with comorbid chronic diseases, and those requiring critical intervention may have immune dysfunction and organ fragility, thereby increasing the risk of SAEs. In addition, the patients in the MIMIC database are mainly from the U.S. population, and their basic health conditions, access to medical care, and the intensity of critical care may be different from those of Chinese hospital patients, which may further affect the probability of SAE occurrence. Different diagnostic criteria used in different studies may also lead to systematic differences in incidence. Some studies use only CAM-ICU as a screening tool, while others combine CAM-ICU with GCS score, the latter of which may provide a more comprehensive assessment in a wider range of high-risk groups (40, 41). Although CAM-ICU can identify delirium, it is mainly used to assess delirium symptoms, which is only one of the clinical manifestations of SAE and fails to cover all SAE-related neurological dysfunctions comprehensively.

SAE has a serious adverse effect on the prognosis of patients with sepsis, but early identification and intervention still face great challenges. Commonly used screening tools (GCS and CAM-ICU) mainly rely on the subjective judgment of medical staff, and the evaluation results are susceptible to bias and lack objectivity and consistency (40, 42). With the deepening of SAE research, the construction of accurate prediction models has become a new research focus. However, among the 10 related studies included in this study, the predictive factors used in each model were quite different, and there is no unified combination of predictive variables. We found that some predictive factors such as age, SOFA score, blood sodium level, platelet count, mean arterial pressure (MAP), and neurobiomarker S100β were repeatedly found in multiple studies, suggesting that they may have stable predictive value in SAE prediction. To further explore the relationship between variable consistency and model performance, we compared the AUC performance of studies using high-frequency variables and similar modeling methods. For models built with logistic regression, the AUC of models that included high-frequency predictors (>2) was mostly higher (>0.8); in machine learning models, high-frequency predictors ranked higher in importance and had larger AUC values. Future studies need to further focus on and verify these potential key variables. However, we included relatively few studies, and the relevant results need to be further verified in more models and populations.

This study found that although the included studies differed in terms of modeling methods and variable selection, most models showed good discriminative performance on their respective data sets. Meta-analysis showed that the combined AUC of the model based on logistic regression was 0.85 (95% CI: 0.77–0.93), suggesting that the existing model has certain potential for clinical application. However, there was high heterogeneity among the studies (I^2^ = 91.8%, *p* < 0.001), and the combined results need to be interpreted with caution. Sensitivity analysis by elimination method showed that heterogeneity may be mainly caused by the study of Jin et al. (36). This may be because the study used the MIMIC database population, which is different from other studies based on Chinese clinical data in terms of characteristic distribution, ethnic composition, modeling sample size, and clinical background of the study population. In addition, the diagnostic criteria for SAE have not been fully unified, and there are certain differences in the assessment tools, judgment time points, and discrimination criteria used in different studies (43). Despite the high heterogeneity, the publication bias assessment results (Egger’s test *p* > 0.05) did not indicate obvious bias. We evaluated the quality of the included models using the PROBAST tool and found that all models exhibited a high risk of bias, which limits their clinical applicability and generalizability. We also referred to the TRIPOD reporting standards and found that most studies did not report sufficiently on key aspects such as predictor selection, missing value processing, and model validation, which may also be an important reason for the high risk of bias. It is recommended that the construction of SAE prediction models in the future should be carried out as much as possible following the standardized development guidelines for prediction models to improve the reproducibility and horizontal comparison capabilities of the models (44).

The prediction models included in this study also have certain positive clinical significance. First, there is the significant role of specific biomarkers. The best-performing logistic model in Zhang et al. (37) combined with neurospecific indicators (cerebrospinal fluid BNIP3L and ONSD), made the model have high accuracy (AUC greater than 0.9) and good clinical benefits. Similarly, Mei et al. (25) used indicators such as cerebral circulation time, S100β, and middle cerebral artery PI to construct a logistic regression model, and the results showed that the model had clinical benefits in the range of 0–100%. This also suggests that compared with simple systemic indicators, combining direct measurement indicators of brain injury or cerebral perfusion can significantly improve the diagnostic accuracy of SAE. However, these studies also have certain problems: both studies were single-center with small sample sizes, and routine cerebrospinal fluid analysis and cranial ultrasound measurements in clinical ICUs are difficult. We also summarized some other routine clinical variables of SAE patients (such as age, SOFA score, MAP, and blood sodium level). These variables not only appear repeatedly in multiple studies but also have good accessibility and ease of application in clinical practice. Future optimization of prediction models should focus on combining special indicators related to brain injury with these routine clinical variables and further improve the accuracy and clinical practicality of early diagnosis of SAE by constructing a multi-dimensional and multi-level prediction framework. In addition, an important consideration is the choice between developing a machine learning model or a traditional logistic regression model. This study found that complex machine learning methods (such as XGBoost and neural networks) can achieve slightly better predictive performance than traditional logistic regression models in the MIMIC dataset. However, this advantage is not absolute. Zhang et al. (37) showed that in the clinical dataset they collected, the neural network was not better than the logistic regression model, and the latter still had good discrimination ability. In general, the selection of predictors and data quality may have a greater impact on model performance than the modeling algorithm itself (45). The logistic regression model has good interpretability and is easy to convert into a clinical evaluation tool, which is easy for clinical medical staff to understand and apply. It still has important clinical value at the current stage. Machine learning methods have unique advantages in dealing with complex feature interactions and nonlinear relationships and are particularly suitable for large-scale and diverse datasets (46). However, its interpretability is poor, and targeted algorithm adjustments are required. It lacks the trust of clinical medical staff, which affects its widespread application in actual clinical settings (47, 48). In the development of SAE prediction models, these two methods can be combined to improve model performance and ensure the interpretability and clinical scalability of the model (49).

All ten studies included in this review were published within the past 5 years, reflecting growing interest in the early prediction of SAE. However, the lack of high-quality external validation remains a key limitation. Future research should focus on developing large-scale, multicenter, prospective models with external validation to assess their stability and clinical utility (50, 51). The high-frequency risk factors summarized in this study can provide a reference for clinical work, and medical staff can strengthen the monitoring and management of high-risk patients in practice. It is important to note that some of the inherent risks identified by the model cannot be directly changed (such as patient age, chronic comorbidities, etc.), so the role of the prediction model focuses more on risk stratification and reminding clinical attention to high-risk groups. Although some of the included models reported higher AUC values (such as above 0.9), it should be pointed out that the improvement in discriminative ability does not mean that the model is reliable in actual clinical applications. All models have the risk of bias in the PROBAST assessment, especially in the analysis domain-related items (such as missing data processing methods and predictor screening strategies). In the future, the construction of SAE prediction models should focus on methodological rigor. We suggest that improvements can be made in the following aspects: (1) There are differences in the definition of SAE. The use of recognized standard definitions will help improve the verifiability and comparability of the model in different data sets; (2) Prospective data collection: More prospective studies are needed to ensure the uniformity of prediction data collection and standardize the outcome evaluation process; (3) Onset time processing: Current studies have not yet clarified which stage of the sepsis course the model is applicable to. In the future, dynamic variables (such as time series or continuous organ dysfunction trajectories) can be considered to improve the prediction ability of SAE, although this will increase the complexity of the model; (4) Variable screening methods urgently need to be standardized and transparent: At present, some studies still only use univariate analysis to screen predictive variables, which may miss important interaction terms or confounding factors, reducing the predictive ability of the model. It is recommended that future studies use more systematic and robust feature selection methods such as LASSO regression, recursive feature elimination, and Boruta algorithm; and (5) Transparency and reporting: Some studies did not clearly report the proportion of missing data, the processing method, and the final form of the model. In addition, the model evaluation indicators are still not comprehensive. Although AUC is a commonly used performance indicator, it is recommended to report indicators such as Brier score, calibration slope, and decision curve analysis (DCA) at the same time to more comprehensively reflect the predictive ability and clinical practicality of the model at different risk thresholds. Future studies should try to follow reporting standards such as TRIPOD to improve research quality and transparency.

This study has some limitations. The number of included studies is relatively limited, and there is high heterogeneity among the studies. The inclusion of only five studies in the meta-analysis may affect the robustness of the results. Although the data summarized in this study came from different regions, the authors of the included studies were all from China, which may have a certain regional bias.

## 5 Conclusion

This study evaluated a variety of SAE prediction models. The results showed that the existing SAE prediction models performed well in terms of discrimination ability, but they all had a high risk of bias and were not recommended for use in current clinical practice. In the future, in the process of model development, it is necessary to pay attention to the quality of the model, reduce the risk of bias, conduct high-quality external validation, and ensure the ease of interpretability of the model.

## Data Availability

The original contributions presented in the study are included in the article/Supplementary material, further inquiries can be directed to the corresponding author.

## References

[1] Gofton TE Young GB. Sepsis-associated encephalopathy. Nat Rev Neurol. (2012) 8:557–66. doi: 10.1038/nrneurol.2012.183, PMID: 22986430

[2] Sonneville R Benghanem S Jeantin L de Montmollin E Doman M Gaudemer A . The spectrum of sepsis-associated encephalopathy: a clinical perspective. Crit Care. (2023) 27:386. doi: 10.1186/s13054-023-04655-8, PMID: 37798769 PMC10552444

[3] Mazeraud A Righy C Bouchereau E Benghanem S Bozza FA Sharshar T. Septic-associated encephalopathy: A comprehensive review. Neurotherapeutics. (2020) 17:392–403. doi: 10.1007/s13311-020-00862-1 32378026 PMC7283452

[4] Liu H Zhang T Zhang L Zhong Y. Neuroinflammatory mechanisms of adult sepsis-associated encephalopathy: implications for blood–brain barrier disruption and oxidative stress. Diagnostics. (2025) 15:873. doi: 10.3390/diagnostics15070873, PMID: 40218223 PMC11988331

[5] Heming N Mazeraud A Verdonk F Bozza FA Chrétien F Sharshar T. Neuroanatomy of sepsis-associated encephalopathy. Crit Care. (2017) 21:65. doi: 10.1186/s13054-017-1643-z, PMID: 28320461 PMC5360026

[6] Zhang SY Zhuo LI Hongguang DING Zhong W Yin WEN Xinqiang LIU . Association of stress hyperglycemia with occurrence and prognosis of sepsis-associated encephalopathy. Chin J Emerg Med. (2023) 32:1070–6. doi: 10.3760/cma.j.issn.1671-0282.2023.08.011

[7] Sonneville R De Montmollin E Poujade J Garrouste-orgeas M Souweine B Darmon M . Potentially modifiable factors contributing to sepsis-associated encephalopathy. Intensive Care Med. (2017) 43:1075–84. doi: 10.1007/s00134-017-4807-z, PMID: 28466149

[8] Lu X Qin M Walline JH Gao Y Yu S Ge Z . Clinical phenotypes of sepsis-associated encephalopathy: a retrospective cohort study. Shock. (2023) 59:583–90. doi: 10.1097/SHK.0000000000002092, PMID: 36821412 PMC10082059

[9] Piva S Bertoni M Gitti N Rasulo FA Latronico N. Neurological complications of sepsis. Curr Opin Crit Care. (2023) 29:75–84. doi: 10.1097/MCC.0000000000001022, PMID: 36794932 PMC9994816

[10] Czempik PF Gąsiorek J Bąk A Krzych ŁJ. Ultrasonic assessment of optic nerve sheath diameter in patients at risk of Sepsis-associated brain dysfunction: a preliminary report. Int J Environ Res Public Health. (2020) 17:3656. doi: 10.3390/ijerph17103656, PMID: 32456003 PMC7277340

[11] Kim TJ Kim J-M Lee JS Park S-H Cha J Bae H-M . Predicting outcomes in patients with sepsis-associated encephalopathy using prefrontal functional connectivity analysis. Sci Rep. (2025) 15:18040. doi: 10.1038/s41598-025-02658-9, PMID: 40410353 PMC12102363

[12] Guo J Cheng H Wang Z Qiao M Li J Lyu J. Factor analysis based on SHapley additive ex planations for sepsis-associated encephalopathy in ICU mortality prediction using XGBoost — a retrospective study based on two large database. Front Neurol. (2023) 14:1290117. doi: 10.3389/fneur.2023.1290117, PMID: 38162445 PMC10755941

[13] Yende S Austin S Rhodes A Finfer S Opal S Thompson T . Long-term quality of life among survivors of severe sepsis: analyses of two international trials. Crit Care Med. (2016) 44:1461–7. doi: 10.1097/CCM.0000000000001658, PMID: 26992066 PMC4949079

[14] Gao Q Hernandes MS. Sepsis-associated encephalopathy and blood-brain barrier dysfunction. Inflammation. (2021) 44:2143–50. doi: 10.1007/s10753-021-01501-3, PMID: 34291398 PMC11044530

[15] Wang R Bi W Huang S Han Q Deng J Wang Z . Recent advances in the pathogenesis, diagnosis, and treatment of sepsis-associated encephalopathy. Brain-X. (2024) 2:e67. doi: 10.1002/brx2.67

[16] Moraes CA Zaverucha-do-Valle C Fleurance R Sharshar T Bozza FA d’Avila JC. Neuroinflammation in Sepsis: molecular pathways of microglia activation. Pharmaceuticals (Basel). (2021) 14:416. doi: 10.3390/ph14050416, PMID: 34062710 PMC8147235

[17] Huang X Wei P Fang C Yu M Yang S Qiu L . Compromised endothelial Wnt/β-catenin signaling mediates the blood-brain barrier disruption and leads to neuroinflammation in endotoxemia. J Neuroinflammation. (2024) 21:265. doi: 10.1186/s12974-024-03261-x, PMID: 39427196 PMC11491032

[18] Miranda F Gonzalez F Plana MN Zamora J Quinn TJ Seron P. Confusion assessment method for the intensive care unit (CAM-ICU) for the diagnosis of delirium in adults in critical care settings. Cochrane Database Syst Rev. (2023) 11:CD013126. doi: 10.1002/14651858.CD013126.pub2, PMID: 37987526 PMC10661047

[19] Chaudhry N Duggal AK. Sepsis associated encephalopathy. Adv Med. (2014) 2014:762320. doi: 10.1155/2014/762320, PMID: 26556425 PMC4590973

[20] Wojtara M Rana E Rahman T Khanna P Singh H. Artificial intelligence in rare disease diagnosis and treatment. Clin Transl Sci. (2023) 16:2106–11. doi: 10.1111/cts.13619, PMID: 37646577 PMC10651639

[21] Fei Y Hao Z Zheng X Ji X Zhao W. Microbiological and clinical predictors of sepsis-associated encephalopathy in bloodstream infections: a retrospective cohort study. Front Cell Infect Microbiol. (2025) 15:1548370. doi: 10.3389/fcimb.2025.1548370, PMID: 40125514 PMC11925888

[22] Yu D Liu J Song X Ao Y Li X Han Y. Analysis of the inflammatory storm response and heparin binding protein levels for the diagnosis and prognosis of sepsis-associated encephalopathy. Eur J Med Res. (2025) 30:116. doi: 10.1186/s40001-025-02369-x, PMID: 39966958 PMC11834667

[23] Zhang Q Zhang X Li Y Zeng L Zhu R Xin Y . Combined cerebral oxygen saturation and neuron-specific enolase evaluation for diagnosis and prognosis of sepsis-associated encephalopathy. Sci Rep. (2025) 15:15369. doi: 10.1038/s41598-025-00353-3, PMID: 40316550 PMC12048628

[24] Mowafy SMS Bauiomy H Kohaf NA Abd Ellatif SE. The role of Ultrasonographic assessment of optic nerve sheath diameter in prediction of Sepsis—associated encephalopathy: prospective observational study. Neurocrit Care. (2025). doi: 10.1007/s12028-024-02187-9, PMID: 39815108 PMC12321661

[25] Mei J Zhang X Sun X Hu L Song Y. Optimizing the prediction of sepsis-associated encephalopathy with cerebral circulation time utilizing a nomogram: a pilot study in the intensive care unit. Front Neurol. (2024) 14:1303075. doi: 10.3389/fneur.2023.1303075, PMID: 38274881 PMC10808420

[26] Han Y Xie X Qiu J Tang Y Song Z Li W . Early prediction of sepsis associated encephalopathy in elderly ICU patients using machine learning models: a retrospective study based on the MIMIC-IV database. Front Cell Infect Microbiol. (2025) 15:1545979. doi: 10.3389/fcimb.2025.1545979, PMID: 40313459 PMC12043699

[27] Moons KGM Wolff RF Riley RD Whiting PF Westwood M Collins GS . PROBAST: a tool to assess risk of Bias and applicability of prediction model studies: explanation and elaboration. Ann Intern Med. (2019) 170:W1–W33. doi: 10.7326/M18-1377, PMID: 30596876

[28] Page MJ McKenzie JE Bossuyt PM Boutron I Hoffmann TC Mulrow CD . The PRISMA 2020 statement: an updated guideline for reporting systematic reviews. BMJ. (2021) 372:n71. doi: 10.1136/bmj.n71 33782057 PMC8005924

[29] Moons KGM de Groot JAH Bouwmeester W Vergouwe Y Mallett S Altman DG . Critical appraisal and data extraction for systematic reviews of prediction modelling studies: the CHARMS checklist. PLoS Med. (2014) 11:e1001744. doi: 10.1371/journal.pmed.1001744, PMID: 25314315 PMC4196729

[30] Zhao L Wang Y Ge Z Zhu H Li Y. Mechanical learning for prediction of Sepsis-associated encephalopathy. Front Comput Neurosci. (2021) 15:739265. doi: 10.3389/fncom.2021.739265, PMID: 34867250 PMC8636425

[31] Ge C Deng F Chen W Ye Z Zhang L Ai Y . Machine learning for early prediction of sepsis-associated acute brain injury. Front Med (Lausanne). (2022) 9:962027. doi: 10.3389/fmed.2022.962027, PMID: 36262275 PMC9575145

[32] Lu X Kang H Zhou D Li Q. Prediction and risk assessment of sepsis-associated encephalopathy in ICU based on interpretable machine learning. Sci Rep. (2022) 12:22621. doi: 10.1038/s41598-022-27134-6, PMID: 36587113 PMC9805434

[33] Zhao Q Xiao J Liu X Liu H. The nomogram to predict the occurrence of sepsis-associated encephalopathy in elderly patients in the intensive care units: a retrospective cohort study. Front Neurol. (2023) 14:1084868. doi: 10.3389/fneur.2023.1084868, PMID: 36816550 PMC9932587

[34] Wang Z Zhao W Chao Y. Establishment and validation of a predictive model for sepsis – associated encephalopathy. Chin J Crit Care Med. (2023) 43:434–9. doi: 10.3969/j.issn.1002-1949.2023.06.002

[35] Zhou H Yuan J Zhang Q Tao J Liu Y. Factors influencing the occurrence of sepsis – related encephalopathy and the construction of a risk model using a column chart. J Difficult Dis. (2023) 22:1245–50. doi: 10.3969/j.issn.1671-6450.2023.12.003

[36] Jin J Yu L Zhou Q Zeng M. Improved prediction of sepsis-associated encephalopathy in intensive care unit sepsis patients with an innovative nomogram tool. Front Neurol. (2024) 15:1344004. doi: 10.3389/fneur.2024.1344004, PMID: 38445262 PMC10912324

[37] Zhang N Xie K Yang F Wang Y Yang X Zhao L. Combining biomarkers of BNIP3 L, S100B, NSE, and accessible measures to predict sepsis-associated encephalopathy: a prospective observational study. Curr Med Res Opin. (2024) 40:575–82. doi: 10.1080/03007995.2024.2322059, PMID: 38385550

[38] Yang Y Liang S Geng J Wang Q Wang P Cao Y . Development of a nomogram to predict 30-day mortality of patients with sepsis-associated encephalopathy: a retrospective cohort study. J Intensive Care. (2020) 8:45. doi: 10.1186/s40560-020-00459-y, PMID: 32637121 PMC7331133

[39] Liu X Niu H Peng J. Enhancing predictions with a stacking ensemble model for ICU mortality risk in patients with sepsis-associated encephalopathy. J Int Med Res. (2024) 52:03000605241239013. doi: 10.1177/03000605241239013 38530021 PMC10966980

[40] Awan OM Buhr RG Kamdar BB. Factors influencing CAM-ICU documentation and inappropriate “unable to assess” responses. Am J Crit Care. (2021) 30:e99–e107. doi: 10.4037/ajcc2021599, PMID: 34719712 PMC8901421

[41] Chung H-Y Wickel J Brunkhorst FM Geis C. Sepsis-associated encephalopathy: from delirium to dementia? J Clin Med. (2020) 9:703. doi: 10.3390/jcm9030703, PMID: 32150970 PMC7141293

[42] Gill MR Reiley DG Green SM. Interrater reliability of Glasgow coma scale scores in the emergency department. Ann Emerg Med. (2004) 43:215–23. doi: 10.1016/s0196-0644(03)00814-x, PMID: 14747811

[43] Zhang Z Guo L Jia L Duo H Shen L Zhao H. Factors contributing to sepsis-associated encephalopathy: a comprehensive systematic review and meta-analysis. Front Med. (2024) 11:1379019. doi: 10.3389/fmed.2024.1379019, PMID: 38835794 PMC11148246

[44] Collins GS Reitsma JB Altman DG Moons KG. Transparent reporting of a multivariable prediction model for individual prognosis or diagnosis (TRIPOD): the TRIPOD statement. BMC Med. (2015) 13:1. doi: 10.1186/s12916-014-0241-z, PMID: 25563062 PMC4284921

[45] Riley RD Pate A Dhiman P Archer L Martin GP Collins GS. Clinical prediction models and the multiverse of madness. BMC Med. (2023) 21:502. doi: 10.1186/s12916-023-03212-y, PMID: 38110939 PMC10729337

[46] Elshawi R Al-Mallah MH Sakr S. On the interpretability of machine learning-based model for predicting hypertension. BMC Med Inform Decis Mak. (2019) 19:146. doi: 10.1186/s12911-019-0874-0, PMID: 31357998 PMC6664803

[47] Kelly CJ Karthikesalingam A Suleyman M Corrado G King D. Key challenges for delivering clinical impact with artificial intelligence. BMC Med. (2019) 17:195. doi: 10.1186/s12916-019-1426-2, PMID: 31665002 PMC6821018

[48] Alkhanbouli R Matar Abdulla Almadhaani H Alhosani F Simsekler MCE. The role of explainable artificial intelligence in disease prediction: a systematic literature review and future research directions. BMC Med Inform Decis Mak. (2025) 25:110. doi: 10.1186/s12911-025-02944-6, PMID: 40038704 PMC11877768

[49] Christodoulou E Ma J Collins GS Steyerberg EW Verbakel JY Calster BV. A systematic review shows no performance benefit of machine learning over logistic regression for clinical prediction models. J Clin Epidemiol. (2019) 110:12–22. doi: 10.1016/j.jclinepi.2019.02.004, PMID: 30763612

[50] van den Boogaard M Pickkers P Slooter AJC Kuiper MA Spronk PE van der Voort PHJ . Development and validation of PRE-DELIRIC (prediction of delirium in ICU patients) delirium prediction model for intensive care patients: observational multicentre study. BMJ. (2012) 344:e420. doi: 10.1136/bmj.e420 22323509 PMC3276486

[51] Wassenaar A van den Boogaard M van Achterberg T Slooter AJC Kuiper MA Hoogendoorn ME . Multinational development and validation of an early prediction model for delirium in ICU patients. Intensive Care Med. (2015) 41:1048–56. doi: 10.1007/s00134-015-3777-2, PMID: 25894620 PMC4477716

