# Supplementary material for: Diagnostic models for sepsis-associated encephalopathy: a comprehensive systematic review and meta-analysis
Source: Front Neurol. 2025 Jul 31;16:1645397. doi: 10.3389/fneur.2025.1645397 (PMC12350483; doi:10.3389/fneur.2025.1645397)
Supplement: Supplementary file 1 [file Data_Sheet_1.zip › Supplementary Material/Scope statement .docx]

**Scope statement**

This systematic review and meta-analysis aimed to evaluate the diagnosis and risk prediction models for sepsis-associated encephalopathy (SAE), a common and dangerous complication in critically ill patients with sepsis. SAE, characterized by acute brain dysfunction without direct central nervous system infection, is associated with increased short- and long-term mortality and significant cognitive sequelae. This study comprehensively synthesizes existing evidence on SAE prediction models, assessing their methodological quality, predictive performance, clinical applicability, and practical utility. By identifying strengths and limitations of current models, this work aims to provide a scientific foundation for the development, optimization, and clinical implementation of robust, interpretable SAE prediction tools.
