# Supplementary material for: Diagnostic models for sepsis-associated encephalopathy: a comprehensive systematic review and meta-analysis
Source: Front Neurol. 2025 Jul 31;16:1645397. doi: 10.3389/fneur.2025.1645397 (PMC12350483; doi:10.3389/fneur.2025.1645397)
Supplement: Supplementary file 1 [file Data_Sheet_1.zip › Supplementary Material/Search strategy.docx]

**
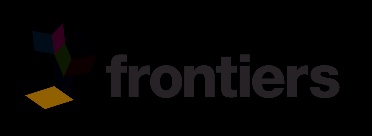
**

***Search strategy***

**The subject terms and free terms were combined to search the Chinese and English databases: China National Knowledge Infrastructure (CNKI), Wanfang Database, VIP Database, SinoMed, Embase, PubMed, Web of Science, Cochrane Library, CINAHL. The details are as follows:**

| **PubMed (n=275)** | |
| --- | --- |
| **#1** | "Sepsis-Associated Encephalopathy"[Mesh] |
| **#2** | ((Associated Encephalopathies, Sepsis [Title/Abstract]) OR (Sepsis-Associated Delirium [Title/Abstract])) OR (Associated Deliriums, Sepsis [Title/Abstract]) |
| **#3** | ("Sepsis-Associated Encephalopathy"[Mesh]) OR (((Associated Encephalopathies, Sepsis [Title/Abstract]) OR (Sepsis-Associated Delirium [Title/Abstract])) OR (Associated Deliriums, Sepsis [Title/Abstract])) |
| **#4** | "Nomograms"[Mesh] |
| **#5** | (((((Partin Nomograms [Title/Abstract]) OR (Partin Tables [Title/Abstract])) OR (Predict [Title/Abstract])) OR (prediction models [Title/Abstract])) OR (risk score [Title/Abstract])) OR (predictors [Title/Abstract]) |
| **#6** | ("Nomograms"[Mesh]) OR ((((((Partin Nomograms [Title/Abstract]) OR (Partin Tables [Title/Abstract])) OR (Predict [Title/Abstract])) OR (prediction models [Title/Abstract])) OR (risk score [Title/Abstract])) OR (predictors [Title/Abstract])) |
| **#7** | (("Sepsis-Associated Encephalopathy"[Mesh]) OR (((Associated Encephalopathies, Sepsis[Title/Abstract]) OR (Sepsis-Associated Delirium[Title/Abstract])) OR (Associated Deliriums, Sepsis[Title/Abstract]))) AND (("Nomograms"[Mesh]) OR ((((((Partin Nomograms[Title/Abstract]) OR (Partin Tables[Title/Abstract])) OR (Predict[Title/Abstract])) OR (prediction models[Title/Abstract])) OR (risk score[Title/Abstract])) OR (predictors[Title/Abstract]))) |

| **Embase (n=72)** | |
| --- | --- |
| **#1** | 'Sepsis associated encephalopathy'/exp |
| **#2** | 'Associated encephalopathies, sepsis':ab,ti OR 'sepsis-associated delirium':ab,ti OR 'associated deliriums, sepsis':ab,ti |
| **#3** | #1 OR #2 |
| **#4** | 'Predictive model'/exp |
| **#5** | 'Nomogram'/exp |
| **#6** | ‘Nomograms':ab,ti OR 'partin tables':ab,ti OR 'predict':ab,ti OR 'risk score':ab,ti OR 'predictors':ab,ti |
| **#7**  **#8** | #4 OR #5 OR #6  **#**3 AND #7 |

| **EBSCO-CINAHL (n=66)** | |
| --- | --- |
| **#1** | AB (Sepsis-Associated Encephalopathy or Associated Encephalopathies, Sepsis or Sepsis-Associated Delirium or Associated Deliriums, Sepsis) AND AB (Nomograms or Partin Nomograms or Partin Tables or Predict or prediction models or risk score or predictors) |

| **Web of Science (n=512)** | |
| --- | --- |
| **#1** | (((TS=(Sepsis-associated encephalopathy)) OR TS=(Associated Encephalopathies, Sepsis)) OR TS=(Sepsis-Associated Delirium)) OR TS=(Associated Deliriums, Sepsis) |
| **#2** | ((((((TS=(Nomograms)) OR TS=(Partin Nomograms)) OR TS=(Partin Tables)) OR TS=(Predict)) OR TS=(prediction models)) OR TS=(risk score)) OR TS=(predictors) |
| **#3** | "#1 AND #2 |

| **Cochrane library (n=47)** | |
| --- | --- |
| **#1** | MeSH descriptor: [Sepsis-Associated Encephalopathy] explode all trees |
| **#2** | (Associated Encephalopathies, Sepsis or Sepsis-Associated Delirium or Associated Deliriums, Sepsis):ti,ab,kw |
| **#3** | #1 OR #2 |
| **#4** | MeSH descriptor: [Nomograms] explode all trees |
| **#5** | (Partin Nomograms or Partin Tables or Predict or prediction models or risk score or predictors):ti,ab,kw |
| **#6**  **#7** | #4 OR #5  **#**3 AND #6 |

| **Chinese database (n=363)** | | **Number** |
| --- | --- | --- |
| **CNKI** | 主题:(脓毒症相关性脑病预 + 脓毒症相关性谵妄 + 败血症精神错乱) and 主题:(预测模型 + 风险模型 + 风险评分 + 模型) | (n = 61) |
| **WangFang**  **VIP**  **CBM** | 摘要:(脓毒症相关性脑病预 or 脓毒症相关性谵妄 or 败血症精神错乱) and 摘要:(预测模型 or 风险模型 or 风险评分 or 模型)  (((摘要=脓毒症相关性脑病 OR 摘要=败血症精神错乱) OR 摘要=脓毒症相关性谵妄) AND (((摘要=预测模型 OR 摘要=风险模型) OR 摘要=模型) OR 摘要=风险评分))  ("预测模型"[常用字段:智能] OR "风险模型"[常用字段:智能] OR "风险评分"[常用字段:智能] OR "模型"[常用字段:智能]) AND (("败血症精神错乱"[常用字段:智能] OR "脓毒症相关性谵妄"[常用字段:智能]) OR ("脓毒症相关性脑病"[不加权:扩展])) | (n = 83)  (n = 107)  (n = 112) |
