# Supplementary figures and images for: Diagnostic models for sepsis-associated encephalopathy: a comprehensive systematic review and meta-analysis
Source: Front Neurol. 2025 Jul 31;16:1645397. doi: 10.3389/fneur.2025.1645397 (PMC12350483; doi:10.3389/fneur.2025.1645397)

# *Supplementary Figures*

- **Supplementary figure:**   **Sensitivity Analysis Fig. 2**


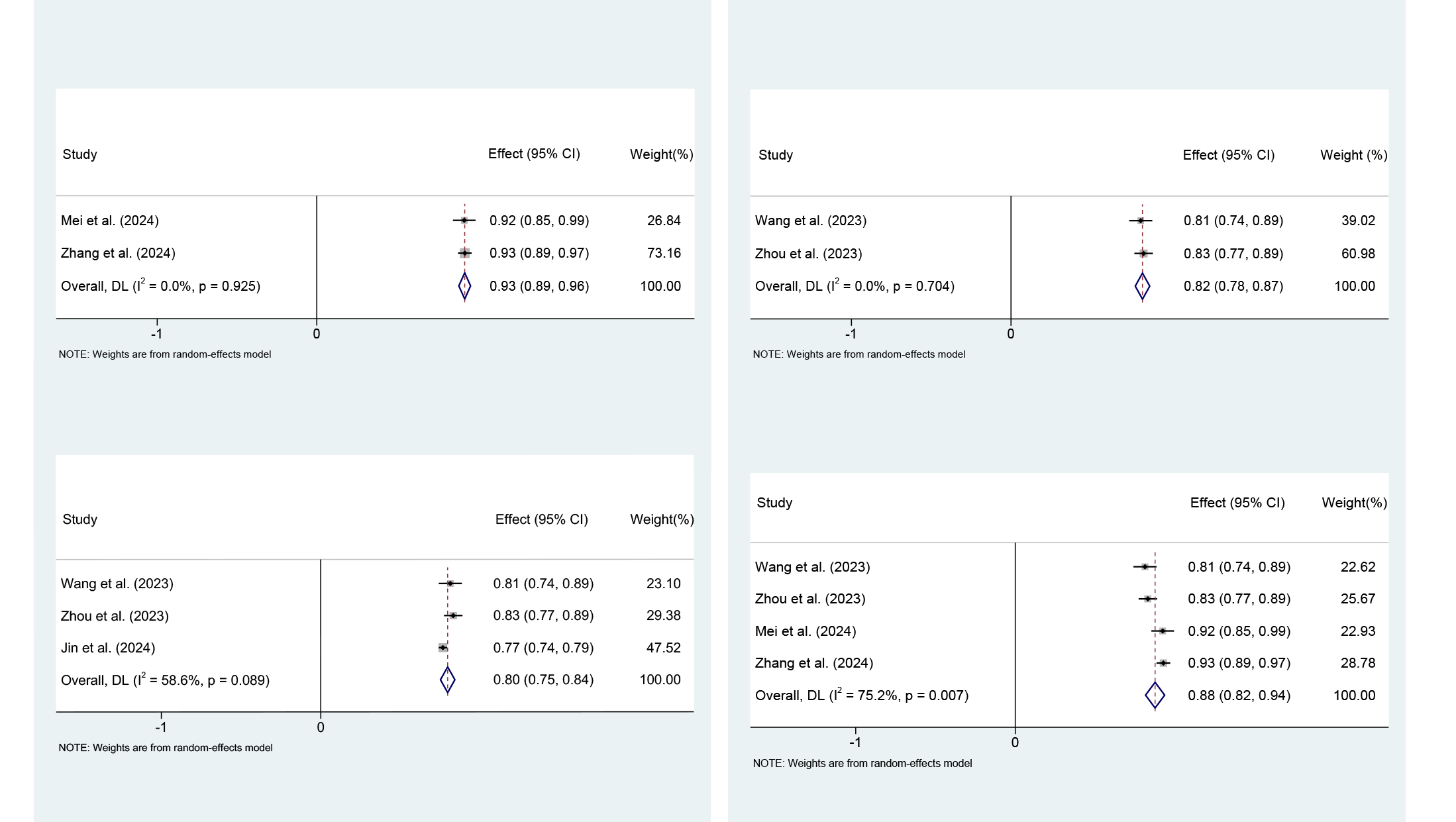

Supplement: Supplementary file 1 [file Data_Sheet_1.zip › Supplementary Material/Supplementary_Figure 1.docx]

# *Supplementary Figures*

**Supplementary figure: Egger’s test result**


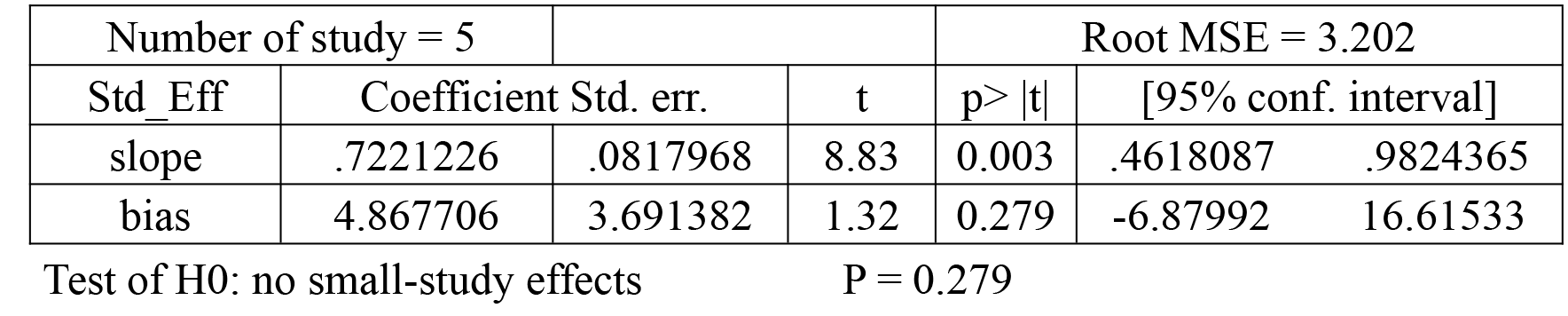

Supplement: Supplementary file 1 [file Data_Sheet_1.zip › Supplementary Material/Supplementary_Figure 2.docx]
